# Genetic Overlap Between Attention Deficit/Hyperactivity Disorder and Autism Spectrum Disorder in *SHANK2* Gene

**DOI:** 10.3389/fnins.2021.649588

**Published:** 2021-04-27

**Authors:** Suk-Ling Ma, Lu Hua Chen, Chi-Chiu Lee, Kelly Y. C. Lai, Se-Fong Hung, Chun-Pan Tang, Ting-Pong Ho, Caroline Shea, Flora Mo, Timothy S. H. Mak, Pak-Chung Sham, Patrick W. L. Leung

**Affiliations:** ^1^Department of Psychiatry, The Chinese University of Hong Kong, Hong Kong, China; ^2^Centre for PanorOmic Sciences - Genomics and Bioinformatics Cores, The University of Hong Kong, Hong Kong, China; ^3^Department of Psychology, The Chinese University of Hong Kong, Hong Kong, China; ^4^Kwai Chung Hospital, Hospital Authority, Hong Kong, China; ^5^Department of Psychiatry, The University of Hong Kong, Hong Kong, China; ^6^Alice Ho Miu Ling Nethersole Hospital, Hospital Authority, Hong Kong, China

**Keywords:** *SHANK* genes, ADHD, ASD, genetic overlap, pleiotropic gene

## Abstract

**Background:** Recent findings indicated a high comorbidity between attention-deficit/hyperactivity disorder (ADHD) and autism spectrum disorder (ASD), as well as shared genetic influences on them. The latter might contribute at least partly to the former clinical scenario. This study aimed at investigating whether *SHANK* genes were potential pleiotropic genes to the two said disorders, underlying their genetic overlap.

**Methods:** This study recruited 298 boys with ADHD (including 256 family trios of 1 ADHD boy and his 2 biological parents), 134 boys with ASD, 109 boys with both ADHD and ASD, and 232 typically developing boys as community controls. They were aged between 6 and 11 years old.

**Results:** There was no significant difference in allele frequency of a number of single nucleotide polymorphisms (SNPs) in *SHANK2*/*SHANK3* between the three clinical groups (ADHD, ASD, and ADHD + ASD) and between the two control groups (community controls and pseudo-controls), respectively. The three clinical groups and the two control groups were thus, respectively, combined. A comparison between the two aggregated samples identified significant evidence of disease association for three *SHANK2* SNPs with both ADHD and ASD, even after multiple testing correction: rs11236616 (*OR* = 0.762, permuted *p* = 0.0376), rs7106631 (*OR* = 0.720, permuted *p* = 0.0034), and rs9888288 (*OR* = 0.770, permuted *p* = 0.0407). Comparisons among individual groups pointed to a similar trend of findings.

**Conclusion:** *SHANK2* could be considered a potential pleiotropic gene underlying the genetic overlap between ADHD and ASD. This might contribute partly to their high comorbidity in the afflicted children.

## Introduction

Attention-deficit/hyperactivity disorder (ADHD) and autism spectrum disorder (ASD) are two separate common neurodevelopmental disorders in children. However, their diagnoses were previously hierarchical such that a diagnosis of ASD precluded a diagnosis of ADHD. The abandonment of this hierarchical diagnostic arrangement in the current psychiatric classification, Diagnostic and Statistical Manual of Mental Disorders-Fifth Edition (DSM-5) (American Psychiatric and Association, 2013) means that the same individual can receive both diagnoses. Subsequent studies have reported high comorbidity rates between the two disorders. For example, 20–50% of ADHD children met diagnostic criteria for ASD, while 30–80% of ASD children also met diagnostic criteria for ADHD (Simonoff et al., 2008; Sinzig et al., 2009). This high comorbidity cannot be explained by diagnostic or phenotypic overlap. The two disorders were found conceptually distinctive and empirically separable by a two-factor common pathway model, while the elevated ASD traits in ADHD children could not be explained by ADHD or other associated behavioral symptoms (Grzadzinski et al., 2011; Ronald et al., 2014). Instead, the high comorbidity of ADHD and ASD is likely to be suggestive of potential sharing of underlying etiologies, including genetic predisposition.

Family-based and twin studies have consistently found that both ADHD and ASD are highly heritable, each with genetic influences accounting for approximately 70–80% of the phenotypic variance and a significant portion of them being shared between the two disorders. The latter was implicated by findings of co-aggregation of ADHD and ASD in the same individuals or in the same families (Reiersen et al., 2008; Mulligan et al., 2009; Ghirardi et al., 2018; Miller et al., 2019). Recently, molecular genetic approaches, such as genome-wide association studies (GWAS), had begun to identify single nucleotide polymorphisms (SNPs) or copy number variants (CNVs) statistically associated with both ASD and ADHD (Rommelse et al., 2010; Martin et al., 2014; Zhao and Nyholt, 2017; Cross-Disorder Group of the Psychiatric Genomics Consortium, 2019). Those genes associated with these implicated SNPs/CNVs are possible pleiotropic genes which contribute to the genetic overlap between ASD and ADHD. Yet, most of the existing studies on ADHD and ASD were conducted at North American and European sites with a relatively small number of ethnic Chinese participants by proportion (Li et al., 2014; Demontis et al., 2019). This present study will be conducted with ethnic Chinese children in Hong Kong.

One strategy for identifying pleiotropic genes for ADHD and ASD with a modest sample size is to take a candidate gene approach, e.g., starting with genes found to be associated with one disorder and testing their association with the other. *SHANK* genes are good candidates for this strategy. They had established association with ASD, reported in both meta-analysis (Leblond et al., 2014) and other independent studies with ASD cohorts and knock-out mice (Peça et al., 2011; Leblond et al., 2014; Connolly et al., 2017). In one animal study, treatment of *SHANK2^–/–^* knock-out mice through restoring NMDA receptor function improved the mice’s ASD traits of impaired social interaction (Won et al., 2012). *SHANK* gene family consists of three members (*SHANK1*, *SHANK2*, and *SHANK3*), which encodes scaffolding proteins required for the proper formation and functioning of neuronal synapses (Monteiro and Feng, 2017). This protein family interacts with a number of synaptic proteins in the postsynaptic density (PSD) of glutamate receptors (NMDA receptors and AMPA receptors). Specifically, SH3 and multiple ankyrin repeat domains protein 2 encoded by *SHANK2*, and SH3 and multiple ankyrin repeat domains protein 3 encoded by *SHANK3* are the two proteins first expressed at the developing PSD. These proteins have been consistently identified to be important for cognitive and emotional well-being, including social behavior (Guilmatre et al., 2014). Thus, there are good theoretical grounds for *SHANK* genes to be associated with ASD.

Though there are fewer reported association between *SHANK* genes and ADHD, our proposition is not entirely without basis. First, paternal balanced translocation of *SHANK3* was reported to result in both ASD and ADHD (Durand et al., 2007; Moessner et al., 2007). Second, *SHANK2^–/–^* knock-out mice displayed both hyperactive and autistic-like behavior (Schmeisser et al., 2012). Based on these lines of existing evidence, the present study aims at testing *SHANK2* and *SHANK3* for pleiotropic effects by examining their common variants (SNPs) for association with both ASD and ADHD.

## Materials and Methods

### Participants

The gender ratios of children with ADHD and children with ASD in the clinic population of Hong Kong were around 10 (male) to 1 (female), as reported in a local study (Yuen, 2017) and in an European study (Nøvik et al., 2006). The marked male preponderance was probably due to detection and referral biases (i.e., fewer girls being detected in the communities and/or referred to clinics), which likely exaggerated the already imbalanced community gender ratios in the eventual clinic population (Rutherford et al., 2016; Young et al., 2020). Thus, only boys were included in this study. Otherwise, having a small number of girls in the sample would make the interpretation of the combined boy/girl results difficult, while separate analysis for gender would prove untenable unless a very huge sample was planned for. Participants consisted of a clinical sample and a community sample. The clinical sample included three groups of boys aged 6–11 years with a primary clinical diagnosis of either ADHD (combined type), ASD or both according to DSM-5: (1) boys with a primary clinical diagnosis of ADHD (combined type) without ASD (ADHD group), (2) boys with a primary clinical diagnosis of ASD without ADHD (ASD group), and (3) boys with dual clinical diagnoses of ADHD and ASD (ADHD + ASD group). Exclusion criteria included the clinical diagnosis of psychosis, bipolar disorder, intellectual disability, Tourette’s or persistent motor or vocal tic disorder, or major neurological or medical illnesses. Operationally, with respect to intellectual disability, children with IQ < 80 were excluded, given potential measurement errors. A community control group of typically developing boys was also recruited. Exclusion criteria, in addition to those for the clinical sample, were parental reports of signs of ADHD, ASD, or other specific learning problems. Research assistants were trained to ask for symptoms of these disorders/problems to parents without using standardized rating scales, as open reports by local parents were generally considered as credible and reliable. This was because local Chinese schooling system was highly demanding both in terms of academic attainment and discipline, the latter including social cooperation among children in group activities, so that children with ASD, ADHD and other specific learning problems were not difficult to be spotted and would be referred for formal assessment in public services. Children whose parents indicated concerns on the above-listed problems or had received queries/complaints/referral for assessment from schools or others on these problems were excluded from the present study.

### Procedure

This study was approved by the Joint Chinese University of Hong Kong-New Territories East Cluster Clinical Research Ethics Committee and complied with the latest version of the Declaration of Helsinki. The clinical sample was recruited from three child psychiatric clinics in Hong Kong. The community control sample was recruited via local mainstream schools or advertisements in social media. The interested parents were first asked two sets of questions to screen whether their children, including the sibling(s) of the child to be participating, had ADHD, ASD, or other specific learning problems. Children, whose parents gave a positive reply to one of the two sets of questions, were declined participation in this study. Parents of the clinical and community control samples provided written informed consent for their children to participate in the study.

Additionally, for the ADHD group, designated funding was available to examine the DNA of the two biological parents for a family-based study of ADHD without the involvement of ASD. Written informed consent was thus obtained from these parents for their own participation. However, this family-based study of ADHD is beyond the scope of this manuscript to be reported here.

### Genomic DNA Extraction

Venous blood was collected from the ADHD group and their biological fathers and mothers. Genomic DNA was isolated using QIAamp DNA Blood Mini Kit (Qiagen, Hilden, Germany) in accordance to a standard protocol. Buccal swabs were collected from the ASD and ADHD + ASD children using Catch-All Buccal Swab (Epicentre Biotechnologies, Madison, United States). Saliva was collected from the community control group using Oragene DNA Collection Kit (DNA Genoteck, Ontario, Canada). Genomic DNA from collected buccal swabs or saliva was isolated by prepIT DNA extraction kit (DNA Genoteck, Ontario, Canada) using a standard procedure.

### SNPs Selection, Genotyping, and Quality Control

Tag SNP strategy was used to select the SNPs in *SHANK2* and *SHANK3* for genotyping. A cluster-based algorithm which clustered SNPs by r^2^ was used (Carlson et al., 2004). The algorithm determined the pairwise r^2^ between all SNPs and clustered correlated SNPs with r^2^ over 0.8 into bins. Each tagSNP selected represented all the SNPs in the same bin. Fifteen tagSNPs for *SHANK2* and eight tagSNPs for *SHANK3* were, respectively, selected by a R^2^ algorithm among SNPs with minor allele frequency (MAF) of at least 10% in Asians (data from HapMap project Phase I- I- I) (Tang et al., 2006). Genotyping was performed using MassARRAY iPLEX platform (Sequenom, San Diego, United States). SNPs with MAF less than 5%, missing rates higher than 10%, Hardy-Weinberg Equilibrium (HWE) *p*-values smaller than 0.1% or above 5% Mendel error rates were excluded. Altogether, three SNPs were excluded with one SNP (rs7103613) in *SHANK2* deviating from HWE distribution (*p* < 0.1%), one SNP (rs13055562) in *SHANK3* having a high missing rate (>10%), and one SNP (rs4980613) in *SHANK2* having a >5% Mendel error rate.

### Statistical Analysis

From the ADHD family trios’ data, we constructed matched pseudo-controls by an in-house R program, which was based on transmitted and non-transmitted parental alleles using the Haplotype-Relative-Risk (HRR) principle (Falk and Rubinstein, 1987). According to this principle, for each SNP locus, the pair of parental non-transmitted alleles was regarded as the genotype of the pseudo-controls. The pseudo-controls could be considered as real random controls and were added to the community typically developing controls to increase the sample size of the controls and thus the statistical power of our significance tests.

The differences in allele frequency between the clinical and control groups were assessed by the χ^2^ test using the PLINK software (Purcell et al., 2007). Haploview v4.2 was employed for visualizing the linkage disequilibrium (LD) pattern. Multiple testing correction was performed by the permutation test with 10,000 times. In this study, permuted <0.05 was regarded as statistically significant. Gene-based association analysis was performed on KGG software (a systemic biological knowledge-based mining system for genome-wide genetic studies), using its GATES function (Li et al., 2010).

## Results

### Participants Recruited

The sample sizes of the three clinical groups were, respectively, 298 boys for the ADHD group (including 256 family trios of 1 ADHD boy and his biological father and mother), 134 boys for the ASD group and 109 boys for the ADHD + ASD group. The community control group had 232 typically developing boys. The mean ages of the ADHD group, ASD group, ADHD + ASD group, and community control group were 9.3 years (*SD* = 1.5), 8.0 years (*SD* = 1.8), 8.5 years (*SD* = 1.4) and 9.2 years (*SD* = 1.7), respectively. There was no significant age difference between the ADHD and control groups, as well as between the ASD and ADHD + ASD groups. However, the former two groups were significantly older than the latter two by about 1 year (*p* = 0.001). Two hundred and fifty-six pseudo-controls were constructed based on 256 family trios’ genotyping data.

### SNP-Level Association Analysis

Thirteen SNPs in *SHANK2* and seven SNPs in *SHANK3* were qualified for data analysis after quality control. Their allele frequency is listed in Table 1. A series of planned hypothesis-driven comparisons were executed. First, we tested whether there were significant differences in the allele frequency between the three clinical groups of ADHD, ASD, and ADHD + ASD, given our hypothesis on *SHANK2/3* as pleiotropic genes, and between the two control groups, i.e., community controls and pseudo-controls, given the latter considered as real random controls. The results showed no significant difference in the allele frequency of the SNPs between the three clinical groups and between the two control groups (Table 1). Given this lack of significant difference among the clinical and control groups, their data were, respectively, merged to form one single aggregated clinical sample to be compared against one single aggregated control sample. This aggregation of groups had the advantage of enlarging the comparison group size and thus increasing statistical power. Assuming an OR of 1.5 and minor allele frequency of 0.4, the statistical power of the comparison between the aggregated clinical sample and the aggregated controls was 90% at a critical *p*-value of 0.001. Before aggregation, the estimated statistical power was, respectively, 72, 36, and 28% for ADHD, ASD, and ADHD + ASD groups vs. the aggregated controls (Genetic Power Calculator)^1^. Significant association with both ADHD and ASD was found for six *SHANK2* SNPs, namely, rs7113016 (*p* = 0.0273, *OR* = 0.819), rs1073294 (*p* = 0.0075, *OR* = 1.275), rs11236616 (*p* = 0.0034, *OR* = 0.762), rs7106631 (*p* = 0.0002, *OR* = 0.720), rs10899158 (*p* = 0.0483, *OR* = 1.194), rs9888288 (*p* = 0.0037, *OR* = 0.770) (Table 1). Three of them survived the multiple testing correction and remained statistically significant (Table 2), i.e., rs11236616 (permuted *p* = 0.0376), rs7106631 (permuted *p* = 0.0034), and rs9888288 (permuted *p* = 0.0407). Significant protective effects were found from the A allele of rs11236616 as well as the T allele of both rs7106631 and rs9888288, all of which decreased disease risk of both ADHD and ASD by around 20–30%. These percentages were calculated based on ORs of those three SNPs. The ORs for rs11236616, rs7106631 and rs9888288 were, respectively, 0.762, 0.720, and 0.770 (Table 2). Assuming approximate equivalence between OR and relative risk for low-frequency diseases, an OR of 0.72 represented a reduction of 0.28 in terms of risk. Thus, ORs between 0.72 and 0.77 represented a risk reduction of around 20–30%. Further genotypic association analysis exhibited even stronger protective effects of rs11236616 (AA vs. AG + GG, *OR* = 0.549, permuted *p* = 0.0376) and rs7106631 (TT vs. TG + GG, *OR* = 0.515, permuted *p* = 0.0013) in a recessive model (Table 2). No significant result was detected for *SHANK3* SNPs by allelic association analysis (Table 1).

**TABLE 1:** Allelic association analysis of SNPs in *SHANK* genes.

**Gene**	**db SNP**	**Physical position**	**Minor/Major allele**	**Minor allele frequency**					***p*-value**							
				**ADHD (*n* = 298) (1)**	**ASD (*n* = 134) (2)**	**ADHD + ASD (*n* = 109) (3)**	**Controls (*n* = 232) (4)**	**Pseudo-controls (*n* = 256) (5)**	**(4) vs. (5)**	**(1) vs. (2) vs. (3)**	**(1, 2, 3) vs. (4, 5)**	**(1) vs. (4, 5)**	**(2) vs. (4, 5)**	**(3) vs. (4, 5)**	**(1, 3) vs. (4, 5)**	**(2, 3) vs. (4, 5)**
*SHANK2*	rs2000605	70353848	T/C	0.463	0.455	0.472	0.439	0.422	0.5751	0.9327	0.1372	0.2031	0.4689	0.2631	0.1357	0.2412
	rs11826745	70376203	C/T	0.405	0.444	0.419	0.416	0.413	0.9343	0.5655	0.8889	0.7136	0.3905	0.8978	0.8090	0.5030
	rs11236570	70379131	A/G	0.416	0.405	0.392	0.421	0.390	0.3278	0.8331	0.8662	0.6726	0.9897	0.7372	0.8394	0.8487
	**rs7113016**	70385037	A/G	0.391	0.371	0.407	0.446	0.430	0.6170	0.7170	**0.0273**	0.0685	0.0534	0.4184	0.0714	0.0700
	rs4550246	70390048	A/C	0.365	0.362	0.362	0.412	0.396	0.6122	0.9941	0.0667	0.1347	0.2200	0.2643	0.0940	0.1288
	**rs1073294**	70401985	G/A	0.448	0.428	0.441	0.400	0.367	0.2933	0.8738	**0.0075**	**0.0118**	0.1919	0.1208	**0.0075**	0.0666
	**rs11236616**	70403442	A/G	0.309	0.308	0.367	0.373	0.390	0.5876	0.2701	**0.0034**	**0.0034**	**0.0276**	0.6841	**0.0112**	0.0770
	**rs7106631**	70405829	T/G	0.385	0.407	0.387	0.457	0.484	0.3980	0.8240	**0.0002**	**0.0009**	0.0722	**0.0312**	**0.0003**	**0.0108**
	**rs10899158**	70405952	T/C	0.460	0.459	0.516	0.438	0.416	0.4917	0.3704	**0.0483**	0.1922	0.3596	**0.0220**	**0.0468**	**0.0433**
	**rs9888288**	70419246	T/A	0.384	0.407	0.458	0.452	0.484	0.3157	0.1678	**0.0037**	**0.0012**	0.0725	0.7786	**0.0065**	0.1601
	rs12363289	70445161	T/C	0.345	0.338	0.257	0.334	0.312	0.4547	0.0534	0.8838	0.2441	0.8724	0.1670	0.1304	0.3451
	rs948191	70452995	G/A	0.325	0.362	0.372	0.336	0.348	0.7032	0.3544	0.9525	0.3632	0.6199	0.0596	0.9550	0.4265
	rs563532	70492408	G/C	0.398	0.432	0.471	0.420	0.437	0.5854	0.1986	0.6325	0.4806	0.5491	0.4119	0.8303	0.3656
*SHANK3*	rs1001469	51138753	A/G	0.411	0.451	0.421	0.405	0.380	0.4289	0.5410	0.1583	0.2215	0.9260	0.3006	0.5366	0.4883
	rs2341011	51139635	T/C	0.325	0.320	0.351	0.344	0.362	0.5615	0.7412	0.2334	0.4662	0.0827	0.4292	0.3591	0.0959
	rs739365	51140316	C/T	0.305	0.267	0.248	0.316	0.287	0.3319	0.2277	0.4095	0.2444	0.2996	0.9279	0.3303	0.4429
	rs2040487	51147015	A/G	0.404	0.410	0.393	0.386	0.390	0.8986	0.9224	0.4743	0.8777	0.2858	0.1253	0.6129	0.0953
	rs5770820	51150473	G/A	0.502	0.485	0.429	0.487	0.494	0.8256	0.1897	0.7306	0.5249	0.5026	0.8992	0.5724	0.5908
	rs6010065	51158017	G/C	0.411	0.399	0.392	0.450	0.415	0.2695	0.8721	0.1992	0.6739	0.8611	0.1022	0.7329	0.2673
	rs8137951	51165664	A/G	0.424	0.428	0.481	0.407	0.449	0.1943	0.3335	0.7299	0.4077	0.3302	0.2822	0.2633	0.1868

*SHANK2, SH3 and multiple ankyrin repeat domains protein 2 gene; SHANK3, SH3 and multiple ankyrin repeat domains protein 3 gene; OR, odds ratio; SNP, single nucleotide polymorphism. Significant SNPs bolded.*

**TABLE 2:** Significant SNPs in *SHANK2* (ADHD/ASD/ADHD + ASD vs. control/pseudo-control).

**db SNP**	**Allele/Genotype**	**Cases/Controls (n, %)**	**Most significant model**	**OR (95% CI)^#^**	***p*-value**	*p*-value**
rs7113016	A*	418 (39)/206 (44)		0.819 (0.686–0.978)	0.0273	0.2479
	G	656 (61)/256 (56)				
	AA	91 (17)/46 (20)	Dominant	0.730 (0.563–0.946)	0.0176	0.1849
	AG	236 (44)/114 (49)				
	GG	210 (39)/71 (31)				
rs1073294	G*	461 (44)/184 (40)		1.275 (1.067–1.524)	0.0075	0.0782
	A	583 (56)/276 (60)				
	GG	90 (17)/36 (16)	Dominant	1.464 (1.124–1.907)	0.0047	0.0541
	GA	281 (54)/112 (49)				
	AA	151 (29)/82 (35)				
**rs11236616**	A*	343 (32)/173 (38)		0.762 (0.635–0.915)	0.0034	**0.0376**
	G	729 (68)/291 (62)				
	AA	44 (8)/32 (14)	Recessive	0.549 (0.368–0.821)	0.0035	**0.0376**
	AG	255 (48)/109 (47)				
	GG	237 (44)/91 (39)				
**rs7106631**	T*	403 (39)/212 (46)		0.720 (0.602–0.860)	0.0002	**0.0034**
	G	629 (61)/252 (54)				
	TT	66 (13)/47 (20)	Recessive	0.515 (0.368–0.721)	0.0001	**0.0013**
	TG	271 (53)/118 (51)				
	GG	179 (34)/67 (29)				
rs10899158	T*	485 (47)/203 (44)		1.194 (1.001–1.425)	0.0483	0.3958
	C	547 (53)/261 (56)				
	TT	104 (20)/43 (19)	Dominant	1.343 (1.022–1.765)	0.0345	0.3244
	TC	277(54)/117 (50)				
	CC	135 (26)/72 (31)				
**rs9888288**	T*	432 (40)/205 (45)		0.770 (0.646–0.919)	0.0037	**0.0407**
	A	636 (60)/249 (55)				
	TT	80 (15)/41 (18)	Recessive	0.639 (0.463–0.883)	0.0067	0.0725
	TA	272 (51)/123 (54)				
	AA	182 (34)/63 (28)				

SHANK2, SH3 and multiple ankyrin repeat domains protein 2 gene; OR, odds ratio; SNP, single nucleotide polymorphism; CI, confidence interval. *Minor allele. **p-value corrected for multiple testing by permutation 10,000 times. Dominant model: homozygous minor allele plus heterozygous vs. homozygous major allele. Recessive model: homozygous minor allele vs. heterozygous plus homozygous major allele. ^#^OR value associated with the minor allele or minor allele genotype of each SNP. Significant SNPs after multiple testing correction bolded.

We then proceeded to finer comparison between groups (Table 1). Each clinical group was individually compared to the combined control sample. We expected fewer instances of significant differences, given the reduced sample size and statistical power (see above power computation). Yet, the overall trend of the results was largely identical to that of the above. Regarding the ADHD group, four of the six above-named SNPs were found to be significantly linked to ADHD with a fifth one (rs7113016) at marginal significance (*p* = 0.0685). The ASD and ADHD + ASD groups had even smaller sample sizes (134 and 109, respectively, as compared to 298 of the ADHD group) and thus reported fewer instances of statistical significance. Although the ASD group reported only one SNP (rs11236616) among the six SNPs identified above to be significantly associated to ASD, there were three more SNPS among them (rs7113016, rs7106631 and rs9888288) reaching marginal significance (*p* < 0.08). The ADHD + ASD group reported two SNPs of significant association to ADHD and ASD among the above six named SNPs (rs7106631 and rs10899158).

Finally, we created two enlarged clinical samples by diagnosis, i.e., adding ADHD and ADHD + ASD groups together to form one enlarged ADHD sample, whilst adding ASD and ADHD + ASD groups to form one enlarged ASD sample. When these two enlarged clinical samples were, respectively, compared to the aggregated control sample, the results came close to the findings involving all three clinical groups combined. For the enlarged ADHD sample, only one of the six above named SNPs (rs7113016) marginally missed the statistical significance (*p* = 0.0714) associated with ADHD. For the enlarged ASD sample (with a smaller sample size of 243 vs. 407 of the ADHD sample), among the above-named six SNPs, two were significantly associated with ASD (rs7106631 and rs10899158) with three more at marginal significance (rs7113016, rs1073294, and rs11236616) (*p* < 0.08) (Table 1).

### Gene-Level Association Analysis

Gene-level association analysis integrates the *p*-values of a number of SNPs within a gene and provides an overall *p*-value for the entire gene. As shown in Table 3, at the gene-level, *SHANK2*, compared to the aggregated control sample, was associated with the ADHD group (*p* = 0.0082), the enlarged ADHD sample (i.e., involving both ADHD and ADHD + ASD groups) (*p* = 0.0034), and finally the aggregated clinical sample (*p* = 0.0031 and permuted *p* = 0.0061). Though not entirely unexpected, the smaller sample size and lower statistical power were unable to pick up the association between *SHANK2* and ASD, represented by the ASD group or the enlarged ASD sample (involving both ASD and ADHD + ASD groups). As expected, no evidence for disease association was identified for *SHANK3*.

**TABLE 3:** Gene-level association analysis.

**Gene name**	**Chromosome position**	**No. of SNPs**	**Gene-based *p-*value**					
			**ADHD vs. control/pseudo-control**	**ASD vs. control/pseudo-control**	**ADHD + ASD vs. control/pseudo-control**	**ADHD/ADHD + ASD vs. control/pseudo-control**	**ASD/ADHD + ASD vs. control/pseudo-control**	**ADHD/ASD/ADHD + ASD vs. control/pseudo-control**
*SHANK2*	GRCh37, chr11:70,313,961–70,935,842	13	**0.0082**	0.2452	0.2252	**0.0034**	0.1175	**0.0031**
*SHANK3*	GRCh37, chr22:51,113,070–51,171,640	7	0.8586	0.4714	0.3508	0.7322	0.2866	0.4759

*SHANK2, SH3 and multiple ankyrin repeat domains protein 2 gene; SHANK3, SH3 and multiple ankyrin repeat domains protein 3 gene; SNP, single nucleotide polymorphism. Significant p-value bolded.*

## Discussion

In terms of the allele frequency of *SHANK2* SNPs, our study finds no significant difference between the three clinical groups, i.e., ADHD, ASD and ADHD + ASD groups, and between the two control groups, respectively. Instead, the three clinical groups combined differ significantly (multiple testing corrected) from the two control groups combined with respect to three *SHANK2* SNPs (rs7106631, rs11236616, and rs9888288). Significant protective effects are found from the A allele of rs11236616 as well as the T allele of both rs7106631 and rs9888288, all of which decrease disease risk of both ADHD and ASD by around 20–30%. Subsequent analysis with each individual clinical group or with the two enlarged clinical samples by diagnosis shows a largely similar trend of findings, despite that the actual results are impacted by the reduced sample size and statistical power of the comparison groups. To the best of the authors’ knowledge, there is no previous study reporting association of *SHANK2* SNPs with both ASD and ADHD. This new finding suggests *SHANK2* to be a potential pleiotropic gene to the two disorders. However, at the gene-level analysis, the comparisons between various ASD groups and the aggregated controls are non-significant (see Table 3). These results may be related to the fact that the *p*-value at the gene-level is an aggregate of *p*-values of the SNPs within a gene. The non-significant SNPs within a gene may mask the significance of a few, particularly when the statistical power is quite low.

**FIGURE 1:**
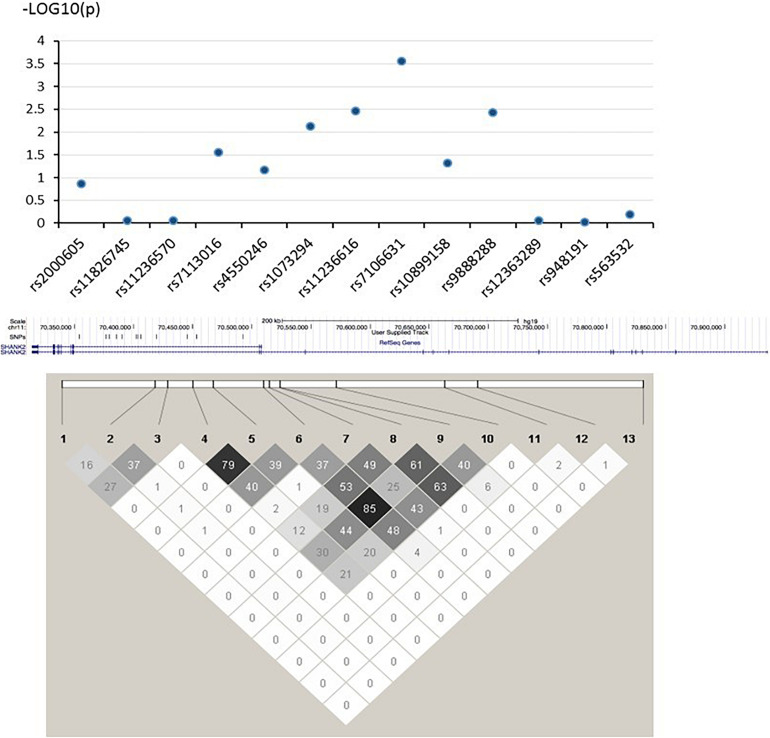
–log (P) graph and pair-wise LD structure of selected SNPs in *SHANK2*. Above graph shows association analysis results of 13 SNPs; each blue dot represents the –log (*p*-value) of each *SHANK2* SNP. Below graph represents the pair-wise LD structure of SNPs in *SHANK2*; the number in each cell is the R^2^ between two SNPs with darker color representing stronger R^2^.

In this study, the three strongest-linked SNPs to ADHD and ASD (rs7106631, rs11236616, and rs9888288) were all clustered in the same intronic region of the *SHANK2* gene (see Figure 1). Although no direct biochemical function could be predicted for three of them, we identified 17 other SNPs which were in LD (*r*^2^ > 0.800) with them using the LD information from the Chinese subpopulation (CHB and CHS) in the 1000G project (The 1000 Genomes Project Consortium, 2015). Three of these 17 SNPs (rs10899152, rs7945377, and rs7111763) were in strong LD with rs7106631 (*r*^2^ = 0.953, 0.857, and 0.841, respectively), the latter being the one SNP identified in this study to be strongest in its association with both ADHD and ASD. Interestingly, the three SNPs associated with this SNP (rs7106631) had been classified as functionally important by RegulomeDB database, affecting transcription factor binding sites or regulating gene expression (Boyle et al., 2012). Thus, this identified SNP of our study, rs7106631, can potentially be involved in the transcriptional regulation or gene expression of *SHANK2* in both ADHD and ASD. Further analysis by expression quantitative trait loci (eQTL), using FUMA^2^, suggested that *SHANK2* rs1893121, which was in strong LD with the currently identified rs7106631 (*r*^2^ = 0.949), was significantly expressed in brain hippocampus (*p* = 0.046) and basal ganglia (*p* = 0.037). Future research should explore and define the functional involvement of our identified SNPs, particularly rs7106631, in these two disorders.

The considerable interrelated nature of psychiatric disorders at the genetic level is speculated to reflect in phenotypic or endophenotypic overlap between disorders, particularly the latter. For example, it would be difficult to find phenotypic overlap between ADHD and schizophrenia; yet, they shared neurocognitive deficits in the endophenotypes of executive functions (The Brainstorm Consortium, 2018). The same possibility may exist for ADHD and ASD. As noted above, their phenotypes were found to be conceptually distinctive and empirically separable (Grzadzinski et al., 2011; Ronald et al., 2014). However, the two disorders were found to share deficits in various components of executive functions, such as working memory, response inhibition, processing speed, fluency and concept formation (Craig et al., 2016; Karalunas et al., 2018). These shared neurocognitive dysfunctions are highly heritable. The involvement of *SHANK* genes in NMDA and AMPA glutamate receptors, which are important in defining synaptic plasticity and cognitive functioning (Guilmatre et al., 2014; Monteiro and Feng, 2017), suggests that *SHANK* genes may play a crucial role in memory and executive dysfunctions found in a wide range of neuropsychiatric disorders, including ADHD and ASD. Thus, one future line of investigation is to examine whether *SHANK* genes represent at least parts of the genetic underpinnings responsible for the development of those shared executive function deficits in ADHD and ASD.

**FIGURE 2:**
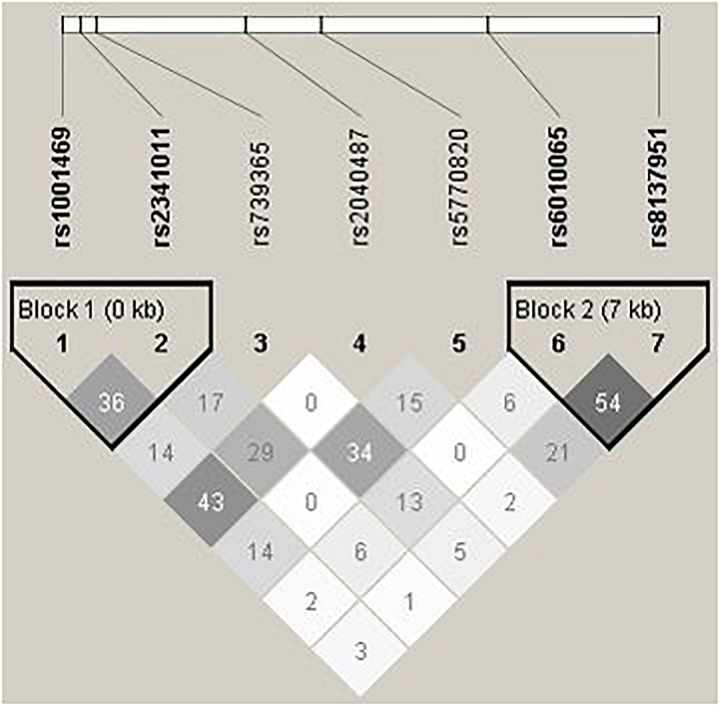
Pair-wise LD structure of selected SNPs in *SHANK3*. The graph represents the pair-wise LD structure of SNPs in *SHANK3*; the number in each cell is the R^2^ between two SNPs with darker color representing stronger R^2^.

On the contrary to *SHANK2*, we do not detect any disease associations with *SHANK3*, whose LD pattern is shown in Figure 2. Previous studies on *SHANK3* with ASD have mainly been conducted with its rare variants (Durand et al., 2007; Moessner et al., 2007; Waga et al., 2011; Boyle et al., 2012; Boccuto et al., 2013; Cochoy et al., 2015; Nemirovsky et al., 2015). Regarding investigation with common variants as in this study, Connolly et al. (2017) explored two ASD family GWAS datasets and found significant maternal genetic effect for *SHANK3* rs5770820 only in the Simons Simplex Consortium dataset, but not in the Autism Genome Project dataset. Furthermore, disease association with *SHANK3* SNPs was also not found or replicated in one Chinese study, which also investigated rs5770820 (Qin et al., 2009). The discrepant findings may be attributable to different study methodology and focus. For example, different from traditional association analysis, the maternal genetic effect of *SHANK3* on ASD detected in Connolly et al.’s study (2017) specifically investigated intrauterine phenotypic development.

In contrast to ASD, *SHANK2* has not been reported to be associated to ADHD in GWAS. In fact, ADHD GWAS have not been very successful in detecting a sizable number of significant SNPs in ADHD until recently by Demontis et al. (2019). This ADHD GWAS in its publication, including supplementary results, reported 12 genome-wide significant loci, but none of them was related to *SHANK2*. However, its sample sizes of 20,183 ADHD participants and 35,191 controls are not considered to be particularly large by comparison to those involved in other common psychiatric disorders, e.g., >450,000 participants in depression (Cross-Disorder Group of the Psychiatric Genomics Consortium, 2019). Thus, the above-cited ADHD sample size may not give sufficient power to identify a larger pool of loci associated with ADHD, which perhaps could include *SHANK2*. Furthermore, as mentioned above, the majority of the participants of these GWAS were from European-ancestry populations with relatively few ethnic Chinese samples by comparison (Li et al., 2014; Demontis et al., 2019).

This study identifies *SHANK2* as the genetic overlap between ADHD and ASD. Yet, there must also be other factors leading to their separate development. Reiersen et al. (2008) found that maternal smoking during pregnancy (SDP) interacted with the 7-repeat allele of dopamine receptor D4 (*DRD4*) gene on the development of ADHD, but not on the development of ASD traits in ADHD children. Interestingly, SDP had been found to be a marker of genetic predisposition than an independent environmental etiological factor of ADHD (Thapar et al., 2009; Skoglund et al., 2014). Indeed, one recent review suggested a genetic overlap between ADHD and SDP (van Amsterdam et al., 2018). Future studies could investigate in more details the shared genes between ADHD and SDP as those contributing to the separate development of ADHD and ASD, in addition to *SHANK2* which contributes to their joint development.

This study has a number of limitations. First, this study only includes boys as participants. It is thus unsure whether our current findings are generalizable to girls. Second, this study relies on clinical diagnosis than on standardized diagnostic interviews. However, while the latter can provide standardization in assessment, clinical diagnosis remains the key criterion against which most, if not all, standardized diagnostic tools are initially validated. Third, in terms of functionality, there are no known functions for those *SHANK2* SNPs which this study identifies. However, we do find our identified SNPs in strong LD with other SNPs involved in the transcriptional regulation or gene expression of *SHANK2*. Fourth, we have only genotyped common variants in the present study; subsequent exome sequencing of this Chinese children sample may uncover potential rare variants which drive the presently observed associations with the *SHANK2* SNPs. Finally, our new finding that *SHANK2* could be a potential pleiotropic gene for ADHD and ASD should be viewed with caution, if not suspicion. Particularly, our modest overall sample size may give rise to potential chance results, despite surviving multiple testing correction. Furthermore, the gene-level analysis is not entirely supportive with respect to ASD. However, even recent large ASD GWAS studies also failed to identify *SHANK* genes (The Autism Spectrum Disorders Working Group of The Psychiatric Genomics Consortium, 2017; Grove et al., 2019), despite the fairly well-replicated association of *SHANK* genes with ASD in previous association and animal studies, cited above (Peça et al., 2011; Won et al., 2012; Leblond et al., 2014; Connolly et al., 2017). Perhaps, these recent ASD GWAS studies are still underpowered, as we are. It is obviously too early to conclude on our new finding and yet dismissing it altogether may not be constructive either. Our study is very much in line with the recent efforts to detect pleiotropic genes and their mechanisms among multiple psychiatric disorders (Cross-Disorder Group of the Psychiatric Genomics Consortium, 2019). These efforts help researchers and clinicians to understand the common phenomenon of high comorbidity among psychiatric disorders. Future study should proceed to confirming our current findings with a much larger independent sample, as well as examining the functional aberrations of our identified SNPs/gene, using both *in vivo* and *in vitro* models.

In clinical practice, screening ADHD in children with ASD and vice versa should be routine. Because of their genetic overlap, they are more likely to be comorbid with each other. The previous hierarchical, mutually exclusive diagnostic practice had seriously misguided clinicians to miss the diagnosis of ADHD in ASD children. Such omission is most unfortunate, since ADHD is a treatable disorder by drug and behavioral intervention. If untreated, it will incur significant psychosocial impairment to a developing child. Our finding also has a significant impact on genetic counseling. In the past, the awareness of the elevated comorbidity between ASD and ADHD was not high. Based on the new knowledge from our study, families seeking genetic counseling will benefit from a better understanding of the genetic interconnectedness of these two disorders in order to make informed decisions.

## Data Availability Statement

The dataset of this study which supports the findings and conclusions of the article will be available from the corresponding authors upon reasonable request.

## Ethics Statement

The studies involving human participants were reviewed and approved by the Joint Chinese University of Hong Kong-New Territories East Cluster Clinical Research Ethics Committee. Written informed consent to participate in this study was provided by the participants’ legal guardian/next of kin.

## Author Contributions

S-LM, C-CL, KL, S-FH, C-PT, T-PH, P-CS, and PL: conceptualization and study design. C-PT, CS, FM, and PL: fieldwork. LC, TM, and P-CS: formal analysis. LC: writing—original draft. S-LM, LC, C-CL, KL S-FH, C-PT, T-PH, CS, FM, TM, P-CS, and PL: writing—review and editing. All authors contributed to the article and approved the submitted version.

## Conflict of Interest

The authors declare that the research was conducted in the absence of any commercial or financial relationships that could be construed as a potential conflict of interest.

## Footnotes

1 https://zzz.bwh.harvard.edu/gpc/

2 https://fuma.ctglab.nl/
